# Mathematics Classroom Assessment: A Framework for Designing Assessment Tasks and Interpreting Students’ Responses

**DOI:** 10.3390/ejihpe11030081

**Published:** 2021-09-18

**Authors:** Eleni Demosthenous, Constantinos Christou, Demetra Pitta-Pantazi

**Affiliations:** Department of Education, University of Cyprus, 1 Panepistimiou Avenue, 2109 Aglantzia, Nicosia, P.O. Box 20537, Nicosia 1678, Cyprus; edchrist@ucy.ac.cy (C.C.); dpitta@ucy.ac.cy (D.P.-P.)

**Keywords:** mathematics classroom assessment, types of mathematics tasks, students’ profiles, instructional adjustments

## Abstract

Classroom assessment could contribute substantially to improving students’ mathematics learning. The process of classroom assessment involves decisions about how to elicit evidence, how to interpret it, and how to use it for teaching and learning. However, the field still needs to further explore how assessment tasks could guide forthcoming instructional adjustments in the mathematics classroom. Towards the endeavor of unpacking the classroom assessment, we present a framework that provides a lens to capture the interplay between the design of mathematics assessment tasks and the analysis of students’ responses. To do so, we relied on existing frameworks of mathematics assessment tasks, and on issues that pertain to the design of tasks. The proposed framework consists of three types of mathematics assessment tasks, their respective competencies, and the characterization of students’ responses. The framework is exemplified with students’ responses from a fourth-grade classroom, and is also used to sketch different students’ profiles. Issues regarding the interpretation of students’ responses and the planning of instructional adjustments are discussed.

## 1. Introduction

### 1.1. Scope of the Paper

Classroom assessment serves as a process for gathering and interpreting evidence to identify students’ progress in order to make decisions about forthcoming actions in day-to-day teaching [1,2]. It would be particularly useful to have lenses that support the interpretation of the evidence elicited from students’ responses in a systematic manner, in order to better understand where students stand on the learning continuum, and in what ways students’ learning could be enhanced. In this paper, we focus on a planned process—that of written assessment tasks. Mathematics tasks differ based on the expected cognitive demand, the mathematical competencies, the thinking processes, the solution strategies, the level of students’ understanding that determine the ways students respond to the tasks, and the kind of information elicited. However, there are limitations in existing frameworks, due to placing less emphasis on the interplay between mathematics assessment tasks and ways of interpreting the evidence elicited from the tasks that could lead to the decision making regarding forthcoming instructional adjustments. Limitations also result from not attending to the bounded classroom context and the descriptive features of students’ work.

Herein, we present a two-dimensional framework that attempts to align the design of assessment tasks with the ways students’ responses could be analyzed. The design of the tasks is intended to elicit information about students’ competencies in mathematics tasks with various levels of contextual familiarity. We also investigate the ways in which students’ responses could be analyzed. The contribution of this framework lies in the identification of a selected competency for each type of mathematics task, which is then used to characterize students’ work. In this way, the framework attempts to characterize descriptive features of students’ work, which are more likely to provide information for effective feedback [3]. We also use empirical data from fourth-grade students to sketch students’ profiles, and then turn to discuss how it would be possible to align these profiles with forthcoming instructional adjustments.

### 1.2. Literature Review

We focus on classroom assessment for formative purposes, using tasks of everyday mathematics to elicit evidence of students’ learning. We then review existing frameworks to identify important components of mathematics assessment tasks. We discuss issues that pertain to the design of assessment tasks and, finally, elaborate on the interpretation of students’ responses and teachers’ actions.

#### 1.2.1. Classroom Assessment

Research suggests that classroom assessment practices for formative purposes have the potential to increase student achievement, and to lead to long-term gains in mathematics performance [1,4]. Particularly, the use of assessment data to individualize instruction has been associated with significant increases in students’ achievement [5]. It has also been found that the speed of learning can be doubled, and the gap between high and low achievers can be reduced [1,6]. Assessment techniques that are embedded within the classroom instruction have also been shown to support teachers in developing better understanding of students’ thinking and misconceptions [7].

Classroom assessment for formative purposes consists of eliciting evidence using instruments that are aligned with instruction and the specific domain, identifying patterns in students’ learning, combining the evidence with general principles to provide meaningful feedback, embedding the assessment into the instructional practice, and designing instructional adjustments [8,9,10,11]. However, less emphasis has been placed on the aspect of instructional adjustments [12]. 

Classroom assessment for formative purposes could range from formative assessment lessons [13] to cognitive diagnostic items [14]. Formative assessment lessons present a rather integrated ongoing approach of assessment [13], while cognitive diagnostic items are intended to provide fine-grained analysis of students’ cognitive knowledge [15]. Classroom assessment practices also include journal reflection, questioning techniques, challenging assignments, assessment tasks, and open-ended performance tasks [16,17]. The various approaches tend to capture students’ learning in order to connect assessment with instruction [18]. The interpretation could be based on the identification of misconceptions, the categorization of students’ strategies, and the quality of students’ arguments. 

Among the various practices for eliciting evidence of students’ current learning, the focus in this paper is on a planned process—that of written assessment tasks. Tasks could provide information about where students stand in terms of learning progression, as well as their levels of understanding [19]. Mathematics tasks for assessment purposes could be used in everyday mathematics teaching, depending on what was taught and what the teacher intends to assess. Tasks should not necessarily form a test, but independent tasks have the potential to provide chunks of information regarding students’ learning in terms of the teaching and learning processes. Empirical results suggest that, within the context of one unit in primary school, it is possible to employ rather extensive formative assessment practices [20]. 

#### 1.2.2. Frameworks for Mathematics Assessment Tasks

We review and analyze frameworks that are relevant to the design and analysis of assessment tasks (Table 1). The first framework, “classroom challenges”, presents four genres of tasks, and was designed to assess and enhance students’ ability to solve multistep, non-routine problems [13]. The second framework presents three levels of thinking [21]. The first framework aligns with a rather radical approach to the classroom culture—that of designing whole lessons of formative assessment—while the second seems to focus more on the design and selection of independent tasks. In this paper, our approach to formative assessment aligns more closely with the design of tasks instead of lessons, as we regard this as an intermediary step along the endeavor of integrating formative assessment in school classrooms. 

We also review frameworks of assessment tasks that are widely used—even in large-scale studies, mainly for summative purposes—to identify important components that need to be taken into consideration. We agree with Thompson et al. that “a given assessment task can be either formative or summative, depending on how the information gathered from that task is used” [22] (p. 4). Harlen also suggests that assessment information could be used “for both summative and formative purposes, without the use for one purpose endangering the effectiveness of use for the other” [23] (p. 215).

Most of these frameworks, including the two aforementioned ones, seem to place emphasis on important mathematical processes, and on procedural and conceptual aspects of mathematical ideas. The MATH taxonomy is a modification of Bloom’s taxonomy for structuring mathematics assessment tasks, and describes the skills that a particular task assesses [24]. Bloom et al. developed a taxonomy for the design and assessment of classroom activities that consist of knowledge, comprehension, application, analysis, synthesis, and evaluation [25]. The TIMSS framework was developed for the purpose of large-scale assessments to compare students’ mathematics achievement in different cognitive domains and content areas, identify trends in students’ performance, and inform evidence-based decisions for improving educational policy and practice across countries over more than 25 years [26]. The MATH taxonomy, TIMSS framework, and de Lange levels seem to have been influenced by Bloom’s taxonomy. Furthermore, the QCAI framework was designed to assess students’ understanding, reasoning, problem solving, and communication in different content areas in order to measure growth in mathematics over time [27]. Finally, the SPUR framework suggests that teachers need to assess understanding of the mathematical content that they teach from four dimensions in order to ensure a balanced perspective in teaching and assessment: algorithms and procedures (skills), underlying principles (properties), applications (uses), and diagrams, pictures, or other visual representations (representation) [28].

**Table 1:** Existing frameworks for assessment tasks.

Framework		Components
1	Problem-Solving Classroom Challenges [13]	Classify and define mathematical objects and structures; represent and translate between mathematical concepts and their representations; justify and/or prove mathematical conjectures, procedures, and connections; identify and analyze structure within situations.
2	Levels of Thinking [21]	Reproduction, procedures, concepts, and definitions; connections and integration for problem solving; mathematization, mathematical thinking, generalization, and insight.
3	MATH Taxonomy [24]	Factual knowledge; comprehension; routine use of procedures; information transfer; application to new situation; justifying and interpreting; implications, conjectures, and comparisons; evaluations.
4	TIMSS [26]	Knowledge of facts, procedures, and concepts; applying the knowledge; reasoning, including analysis, evaluation, generalization, and problem solving.
5	QUASAR Cognitive Assessment Instrument (QCAI) [27]	Understanding and representing mathematical problems; discerning mathematical relationships; organizing information; using strategies, procedures, and heuristic processes; formulating conjectures; evaluating the reasonableness of answers; generalizing results; justifying answers or procedures; communicating mathematical ideas to reflect the complex construct domain of mathematical problem solving; reasoning and communication.
6	SPUR [28]	Skills, properties, uses, and representations.

The first five frameworks attempt to highlight the nature of mathematics by incorporating important mathematical processes such as problem solving, reasoning and proof, communication, connections, and representation. The last five frameworks refer to the application of procedures or skills in various ways to mathematical concepts or relationships. The second, third, and fourth frameworks more clearly incorporate the idea of assessing students from reproduction to application, and then to mathematical reasoning.

The categorization of tasks in the frameworks above is informative about the kinds of processes that students would need to engage with throughout the assessment tasks. However, the mere categorization into the types of knowledge or processes presents limitations when it comes to how a classroom teacher could be informed about their students’ learning during a series of lessons on a mathematical idea. Assessment for formative purposes is administered according to students’ needs, and is closely associated with the curriculum [29]. Sociocognitive and sociocultural theories also seem more suitable for classroom assessment—particularly for achieving alignment between the curriculum and the classroom instruction [29]. Hence, the proposed framework in this paper relies on these existing frameworks, but also attempts to move a step further by making links between the processes and the interpretation of students’ responses. We aimed for a framework that sheds light on students’ emergent, robust, or even fragmented understanding as they engage with mathematical ideas within a classroom community.

#### 1.2.3. Design of Assessment Tasks

The assessment tasks should be meaningful and worthwhile opportunities to learn, as well as being accessible to students [30]. They should drive classroom learning activities and indicate what kinds of instruction should be encouraged [28,30,31]. Tasks that are intended to elicit students’ thinking are usually longer than typical tasks—such as multiple-choice tasks—and take more time to complete, since they engage students with a higher cognitive load [31]. It is inevitable that different types of tasks provide different types of evidence regarding students’ understanding. Shorter tasks could be used to provide instant feedback to the teacher about students’ understanding, while longer tasks could provide insight into students’ thinking, and opportunities for classroom discussions.

Students’ previous experiences and familiarity with the mathematical idea(s) being assessed could change the expected student processes [32]. Students tend to solve tasks that share critical properties with textbooks’ tasks by recalling facts and procedures, while they use creative reasoning for those tasks that do not share those critical properties [33]. The structure, with respect to the level of openness of the task, is another element to be taken into consideration [34]. Structuring the task into successive parts lowers its intended demand [31]. The amount and complexity of textual and visual information, such as the use of terminology and complex sentences, increase students’ reading load [35]. The complexity of the task could also be determined by the number of steps and variables [31]. Overall, the way the language is used, as well as the forms of the questions in the tasks, relate to how students engage with them [34].

Another issue is the context in which the tasks are framed. On the one hand, the context could make the task accessible to students, and give them latitude to display what they know [30]; on the other hand, the context creates challenges in students’ engagement and in the decision making of the task design (e.g., whether the context plays critical role in the mathematization process) [21]. These design issues also moderate the feedback that the teacher receives based on students’ engagement with the task. However, the way in which this happens is poorly understood [6].

#### 1.2.4. Interpretation of Students’ Responses and Teachers’ Actions

The analysis of students’ responses in mathematics assessment tasks needs further study in order to lead to meaningful insight that informs teachers about forthcoming instructional adjustments. Pellegrino, Chudowsky, and Glaser mention that “cognition, observation, and interpretation must be explicitly connected and designed as a coordinated whole. If not, the meaningfulness of inferences drawn from the assessment will be compromised” [36] (p. 2). Indeed, “good teaching decisions are based on high-quality information” [37] (p. 100). 

Analytic rubrics could be used to interpret students’ responses in tasks, which result in identifying elements that should be included in the response [37]. Another approach is the use of holistic rubrics, in which the overall quality of students’ work is assigned to predetermined categories [37]. For example, rubrics have been used to support teachers and students to provide feedback for students’ competencies, and to help both understand the competencies required [38]. Rubrics support the feedback process which, in turn, seems to have a major impact on students’ learning [39].

Teachers who have a better understanding of the learning goals might design richer learning experiences, be more prepared to provide effective formative feedback, and plan remediation instruction. Teachers’ forthcoming adjustments based on elicited evidence could include immediate modification of instructional decisions, planning instructional activities, diagnosing learning difficulties, placing students into learning sequences, recording for later use, and even eliciting further evidence [37]. Teachers need to know how to ensure that the inferences made from assessment tasks are of sufficient quality to understand where the learner is along the learning continuum, and to inform decisions about the next instructional steps to be taken [40]. 

Instructional actions that are effective in supporting students’ learning of procedures and skills would differ from those that are appropriate for developing students’ understanding and sense-making [41]. Tasks with a lower level of challenge may help students to engage easily with classroom activities, as may tasks with multiple representations or solving processes [42]. Moreover, too many challenging tasks in a limited time may demotivate students, even if such tasks promote mathematical reasoning [42]. The types of tasks, the variation in challenge level, and the timing are issues to be considered when planning instructional adjustments to support the learning of mathematics.

#### 1.2.5. Aims of the Paper

The purpose of this paper is twofold: first, it aims to present a framework, and second, to examine its application for classroom assessment. The framework provides a tool and the relevant language for designing mathematics assessment tasks and analyzing students’ responses to them. The framework is exemplified with students’ actual responses in assessment tasks in order to develop insight into how the framework could be employed to explore students’ learning of the mathematical idea(s) under study. To do so, we sketch students’ profiles, and then use the framework to set the grounds for making hypotheses for further instructional adjustments.

## 2. Method

### 2.1. Proposed Framework

The framework is presented in Table 2; it aligns the design of mathematics assessment tasks with the analysis of students’ responses. The first column presents the names of the three types of tasks—reproduction, application, and generation and reflection tasks. In the second column, we refer to the mathematical processes that students are expected to engage with. These processes are partially determined by the contextual familiarity, which is presented in the third column of the table. The contextual familiarity relies on the previous teaching and learning experiences in the classroom, which are known to the classroom teacher. Students’ responses in each type of task are analyzed through a selected competency shown in the fourth column. Then, students’ responses are characterized using the descriptions presented in the fifth column.

#### 2.1.1. Expected Processes

Several of the frameworks presented in Table 1 appear to agree in assessing students from reproduction tasks to higher level thinking tasks. We relied on the categorization of the task processes in these frameworks to define the expected processes for the three types of tasks, and then further refined these processes with reference to the contextual familiarity. Herein, each process is described according to how students are expected to engage with mathematical ideas. Hence, the processes are operationalized with consideration of the previous teaching and learning experiences in the classroom. Mathematical ideas include facts, rules, definitions, and procedures. 

In reproduction tasks, students are expected to rely on recalling mathematical ideas. The minimum requirement is reliance on memory, since the contextual familiarity is that students have had extensive practice with these mathematical ideas (e.g., repeating the same definition in classroom, practicing the multiplication tables). Students may respond not only by reproducing, but also by reconstructing mathematical ideas. Such tasks are part of everyday mathematics teaching, and could inform the teacher whether students are able to respond to tasks that they have practiced extensively.

In application tasks, students are expected to apply mathematical ideas. It does not suffice for students to reproduce taught ideas; they need to decide which mathematical ideas to use, and in what way to use them, according to the format of the task. In detail, the variation in the format of the task creates the need to make inferences and adjust the taught mathematical ideas accordingly. 

In generation and reflection tasks, students are expected to reflect on mathematical ideas and generate arguments, justifications, strategies, and models. In such a task, “it requires a process of stepping back and reflection on the information being connected” [43] (p. 5). Students need to decide not only how to adjust the mathematical ideas to the format of the task, but also how to make sense of the structure of mathematics.

Identifying tasks that correspond to these three types of processes relies on the expected formulation of the tasks. For example, a reproduction task for second-grade students might be an application task for first-grade students. An application task might also engage students in reproducing a known algorithm. Hence, we relied on identifying the expected processes by modifying the approach of the “expected formulation of tasks” for the case of assessment tasks. “The expected formulation of a task represents the path the students in a particular classroom community are anticipated to follow if their community engaged with the task in the ways designed in the curricular resource from which the task was derived” [44] (p. 70). For the case of assessment tasks, the expected formulation of a task relies on the path that students are anticipated to follow based on what preceded in the lesson plan, and the curriculum materials used in the classroom. 

#### 2.1.2. Contextual Familiarity

Mathematical knowledge is developed through the personal (mental) and the institutional (contextual) dimensions [45]. Hence, the assessment needs to be relevant to the context in which the student participates [46]. We delineate the adaptation of the “expectation formulation” of assessment tasks by focusing on the contextual familiarity, and in particular determining how familiar the format of the task is, as well as the work procedure to complete the task. We relied only on students’ prior experiences in the classroom, which are known to the teacher, and acknowledge the limitation that students have further experiences from prior grades and the home environment. Since the framework also aims to become a tool for classroom teachers, we focused on a rather simple categorization of the format and the work procedure as “familiar” or “unfamiliar”.

The format of the task refers to how the request of the task is presented, and how the information is given. The format could change due to variation in representations, scenarios, the number of steps, or examples of numbers/shapes. The work procedure refers to the steps for completing the task. In reproduction tasks, both the format and the work procedure are expected to be familiar. The familiarity results from extensive opportunities for practice. In application tasks, the format is expected to be unfamiliar, while the work procedure is expected to be familiar. Thus, students need to identify how to use the taught mathematical idea(s) in an unfamiliar format, but afterwards, the procedure to complete the task is expected to be familiar. The unfamiliar format needs to be substantially different, often in a nuanced way, depending on the mathematical idea(s) under study (e.g., relying on students’ common misconceptions). In generation and reflections tasks, both the format and the work procedure are expected to be unfamiliar. Hence, students not only need to interpret and identify what kind of taught mathematical idea is relevant to the task, but they also need to construct a series of steps to reach a conclusion.

#### 2.1.3. Competency

In the context of this study, we defined as a competency per type of task the mechanism that acts as a lens to analyze students’ responses. In mathematics education, there is great consensus that students need to engage in representation, reasoning and proving, communication, problem solving, generalization, making connections, and modelling [27,47]. These are called processes, practices, or competencies [48], and also appear in the majority of the frameworks presented in Table 1. However, for the purpose of classroom assessment, we identified constraints in identifying, for example, at which point of the learning continuum a student is at problem solving for a taught mathematical idea (e.g., addition of fractions). Another constraint was that communication and representation, for example, could be seen as media that convey students’ thinking as identified in different types of tasks. Furthermore, we aimed to identify competencies that could be applicable to a range of mathematical topics for primary mathematics, and could also be used for the characterization of students’ responses. The selected competencies for this framework are fluency, flexibility, and reasoning (fourth column in Table 2). 

For reproduction tasks, the teacher would intend to explore how fluent the student is in recalling taught mathematical ideas, considering their extent of practice and familiarity with the task. For application tasks, the focus is on students’ flexibility, as the teacher would intend to elicit how students’ mathematical ideas are adapted, related, kept coherent, and “freed from specific contexts” [43] (p. 3) in various task formats. Generation and reflection tasks turn the focus to students’ reasoning. Reasoning is a common term in mathematics education, often having a meaning close to thinking. Here, reasoning is the production of assertions and justified inferences to reach conclusions using, for example, deductive, inductive, and abductive processes [49].

#### 2.1.4. Characterization of Students’ Responses

Based on the selected competency for each type of task, the framework presents characterizations of students’ responses. The characterization relies on snapshots of aspects of students’ learning being assessed in the assessment tasks (Table 3). The evidence from students’ responses to a reproduction task could indicate developed fluency, developing fluency, or limited fluency. In the same way, the evidence from an application task indicates developed flexibility, developing flexibility, or limited flexibility, while evidence from a generation and reflection task could suggest developed reasoning, developing reasoning, or limited reasoning.

The framework could be viewed horizontally and vertically in a dynamic fashion. Students’ responses could be compared along the continuum in order to identify how students respond to the same tasks (vertical interpretation). Hence, the teacher could decide on how the whole class performs to the processes of different tasks. Students’ responses could also be used to describe their profiles (horizontal interpretation). Hence, the teacher could decide on what instructional adjustments are most appropriate for each student.

### 2.2. Development of the Framework

The development of the framework started with the analysis of existing frameworks, and the mathematical ideas under study, by examining the mathematical standards in the curriculum, the terminology, the expected representations, and students’ common misconceptions. Students’ familiarity with the tasks was determined by exploring the types of tasks found in textbooks—since teachers and students rely extensively on the unique textbook series used in all state schools in the educational context under study—as well as teachers’ lesson plans when these were available [50]. We also explored the content quality by looking at whether the content was sufficiently consistent with the current priorities of the field of mathematics education in order for the tasks to be worthwhile [51]. We also discussed with mathematics education experts what kinds of evidence each assessment task was meant to elicit. Two mathematics educators, who are experienced in the design of tasks for primary mathematics, advised us on the design and analysis of tasks. Then, we turned our attention to the task features and the specification of the tasks by considering issues that pertain to the design of tasks for classroom assessment. Further on, we implemented the assessment tasks and piloted the characterization of students’ responses. We administered 161 tasks to 5 classrooms from grade 4 to grade 6 over the course of a whole school year. The assessment tasks were administered in collaboration with the classroom teachers when the mathematical ideas assessed in the tasks were taught in the respective classrooms. Students solved the tasks independently. We then analyzed students’ responses to explore whether their responses revealed the expected processes [51]. Herein, we present the final version of the framework, and empirical data from one classroom, to illustrate the application of the framework for classroom assessment. 

### 2.3. Design of Assessment Tasks

We exemplify the framework with assessment tasks on multidigit multiplication, and discuss the analysis of students’ responses. The origin of multiplication is based on repeated addition and the schema of correspondences [52]; it is a binary operation with two distinctive inputs, and students need to coordinate the multiplicand (number of elements in each set) and the multiplier (number of such sets), along with the procedure to find the product [53]. 

Multidigit multiplication includes a series of steps for finding the product, and relies on extending single-digit multiplication [54]. Students need to achieve two coordinations: the first coordination is between the magnitudes of factors and the magnitudes of products, while the second coordination is between the expanded forms of factors and the distributive property [54]. Multiplication methods rely on multiplying digits—either manipulating the digits in their expanded form (e.g., 3 in 36 as 3 tens, or 30) or manipulating them as single digits. Particularly, students’ understanding of the distributive property prepares them for finding the product in multidigit multiplication [55] in fractions and algebra [54]. The different types of situations that involve multiplication are equal groups, multiplicative comparison, Cartesian product, and rectangular area [56]. 

The assessment tasks for multidigit multiplication were designed and selected based on the contextual familiarity. The decisions were based primarily on the examination of textbooks, since the teaching approach depicted in textbooks is anticipated to be the dominant one in classrooms since teachers, in the educational context in this study, rely heavily on textbooks for planning and implementing their lessons [50]. In fourth-grade textbooks, the lessons begin with how to use single-digit multiplications to find multidigit multiplications in which one of the factors is a multiple of 10, using the commutative and associative properties. Then, attention turns towards strategies for estimating the product. Afterwards, rectangular arrays are used to find the product of two- and three-digit numbers with one-digit numbers. This approach is then linked with the distributive property of multiplication over addition and subtraction. Finally, the lessons probe students to explore different forms of vertical algorithms (e.g., expanded forms and shorter forms), before reaching the standard algorithm. Below, we present four assessment tasks.

**Figure 1:**
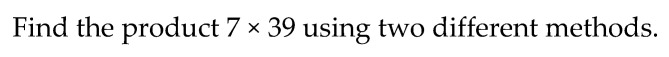
Reproduction task.

The “reproduction task” (RT) is shown in Figure 1; it explores whether students could reproduce two different methods to find the product of a two-digit number by one-digit numbers. Both the standard algorithm and the use of the distributive property are expected to have been taught and practiced beforehand in the classroom. Hence, students are anticipated to have extensive familiarity with the format of the task and the work procedure.

**Figure 2:**
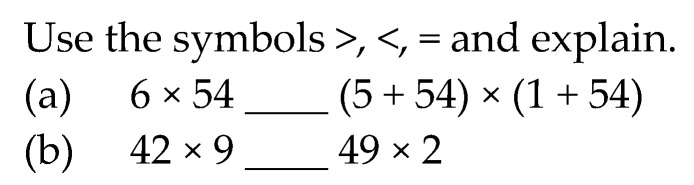
Application task 1.

The first “application task” (AT1) is shown in Figure 2. The task intends to engage students in comparing mathematical expressions in different forms (e.g., varying the place of addition and multiplication symbols, the place of digits). The first set of expressions intends to explore whether students understand the distributive property, and whether they would consider the expression (5 + 54) × (1 + 54) as equivalent to 6 × 54. The second set of expressions intends to investigate whether students would inappropriately apply the commutative property by ignoring the place value of numbers. Students are asked to explain their rationale in order to provide further insight into their thinking.

**Figure 3:**
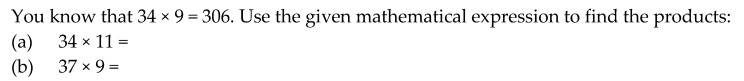
Application task 2.

The second “application task” (AT2) is shown in Figure 3. The task asks students to use the given information (i.e., 34 × 9 = 306) to find the product in the other expressions, where either the multiplier or the multiplicand differs. The task intends to engage students in adjusting the procedure of the distributive property, since they are asked not to analyze one of the factors in tens and units, but to analyze them according to the given information. In the two application tasks, the work procedure is familiar, but the format of the tasks is unfamiliar, since they have to interpret the given information carefully and adapt the known algorithms.

**Figure 4:**
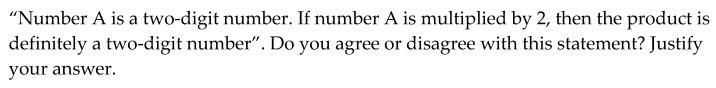
Generation and reflection task.

The “generation and reflection task” (GRT) is shown in Figure 4. Students are asked to form an argument to justify whether they agree or disagree with the given statement by exploring how the numerical structure of the factors relates to the product. They are expected to reflect on the structure of the numbers, and to find a counterexample. They also need to verbalize their argument. The format of task is unfamiliar, as is the work procedure, since students need to decide how to work in order to justify an answer.

## 3. Results

We present the analysis of students’ responses from one fourth-grade classroom with 21 students in order to exemplify the application of the framework for classroom assessment. We elaborate on the process of analysis, as well as the vertical and horizontal perspectives of the framework.

### 3.1. Process of Analysis

The analysis of students’ responses followed two stages: (1) one researcher used the characterizations to code the students’ work; (2) the other two researchers independently coded a sample of students’ responses. Any discrepancies were discussed with the whole group of researchers until consensus was reached.

### 3.2. Vertical Perspective

The vertical perspective of the framework provides an overall picture of the classroom (Table 4). The analysis suggests that the majority of the students have developed fluency in using the procedure for finding the product. However, the class needs to work further on adapting the procedure to different formats, since 16 students showed limited or developing flexibility. The majority of students also showed limited reasoning. Hence, the results suggest that the teaching needs to focus on instructional actions to enhance students’ flexibility and reasoning.

### 3.3. Horizontal Perspective

**Figure 5:**
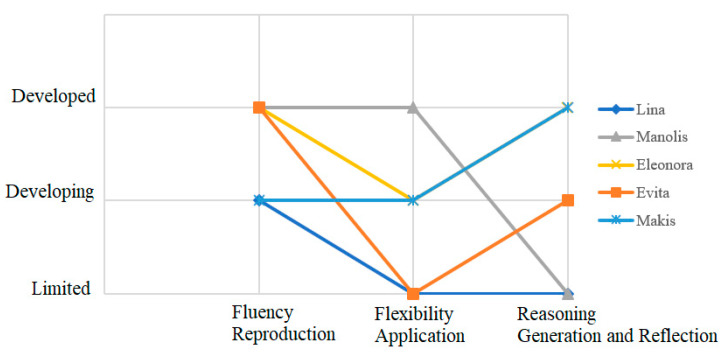
Students’ profiles.

We also elaborate on the horizontal perspective of the framework by presenting selected students’ profiles—namely, the profiles of Lina, Manolis, Eleonora, Evita, and Makis (Figure 5). The selection aimed to (1) illustrate all of the different characterizations (presented in Table 4) by relying on students’ responses (i.e., developing and developed fluency; limited, developing, and developed flexibility; and limited, developing, and developed reasoning), and (2) reveal different profiles of students according to how they responded across the tasks. For example, Lina showed developing fluency, and limited flexibility and reasoning, while Makis also showed developing fluency, but developing flexibility and developed reasoning. In this way, it is then possible to compare different students’ profiles, and to use the profiles as cases for discussing instructional adjustments.

**Figure 6:**
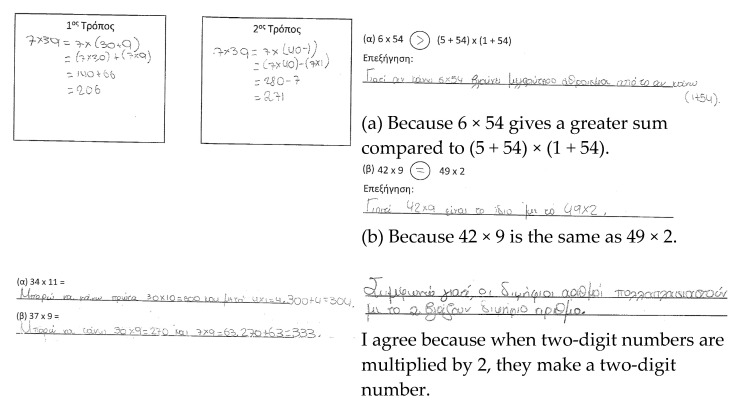
Lina’s responses.

Lina. Lina used the taught procedures in the RT, but made computational mistakes when using the distributive property of multiplication over addition and subtraction (Figure 6). It was not possible for her to apply the procedure of multiplication in the ATs. Lina mentioned that 6 × 54 was greater than (5 + 54) × (1 + 54), without converting the second expression into a comparable form (e.g., 59 × 55) to the first one. Similarly, Lina did not use the procedure flexibly to compare the expressions 42 × 9 and 49 × 2. In the AT2, Lina applied the distributive property by splitting the number into tens and digits, without adapting the procedure flexibly based on the given product. In the GRT, Lina mentioned that the product of a two-digit number by 2 is a two-digit number. Lina is developing fluency, but limited flexibility and reasoning are evident in these four tasks for the concept of multiplication under study. In total, seven students had the same profile as Lina.

**Figure 7:**
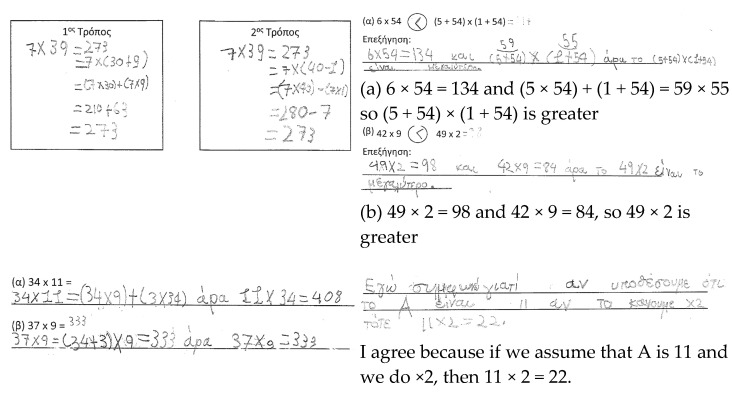
Manolis’ responses.

Manolis. Manolis showed a systematic method of work in the RT (Figure 7). In the AT1, he compared the multiplicands in the first set of expressions, while he tried to find the product in the latter set to compare the expressions. He relied more on the taught procedure than on the magnitude of the numbers (i.e., 4 tens times 9 compared to 4 tens times 2), and made computational mistakes. In the AT2, he adjusted the taught procedure to the context of the task by analyzing the multiplicands based on the given information. In the GRT, Manolis agreed with the given statement, and gave an example to justify his answer. He did not explore the whole spectrum of two-digit numbers to refute the statement. Manolis showed developed fluency and flexibility, while his mathematical reasoning was limited.

**Figure 8:**
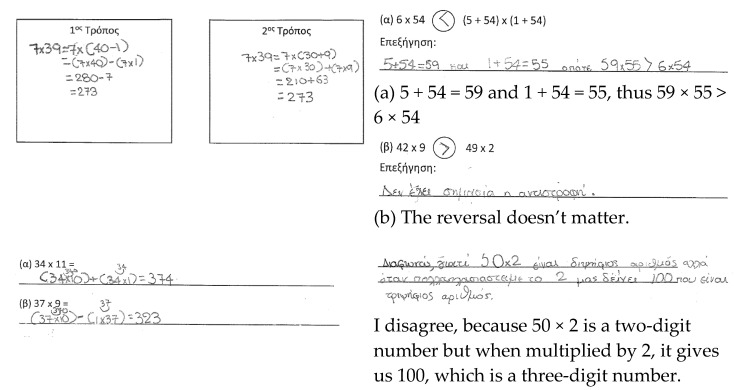
Eleonora’s responses.

Eleonora. Eleonora also used the taught procedure fluently in the RT (Figure 8). In the AT1, she decided that 59 × 55 is bigger than 6 × 54, without finding the product. In the latter set of expressions, Eleonora decided that the change in the place of numbers does not matter, and said that 42 × 9 is greater than 49 × 2. Even though she adapted the procedure flexibly to respond to the AT1, this was not the case in the AT2, in which she found the product by analyzing the number in tens and units, without considering the given information (i.e., students were anticipated to split 11 into 9 + 2). She either did not consider the given statement, or she faced difficulties in extending her current method of using the procedure of distributive property to find the product. In the GRT, Eleonora found that the double of 50 is a three-digit number, thus presenting a counterexample to refute the argument. Hence, Eleonora showed developed fluency and reasoning, and developing flexibility in the four tasks. In total, four students had the same profile as Eleonora.

**Figure 9:**
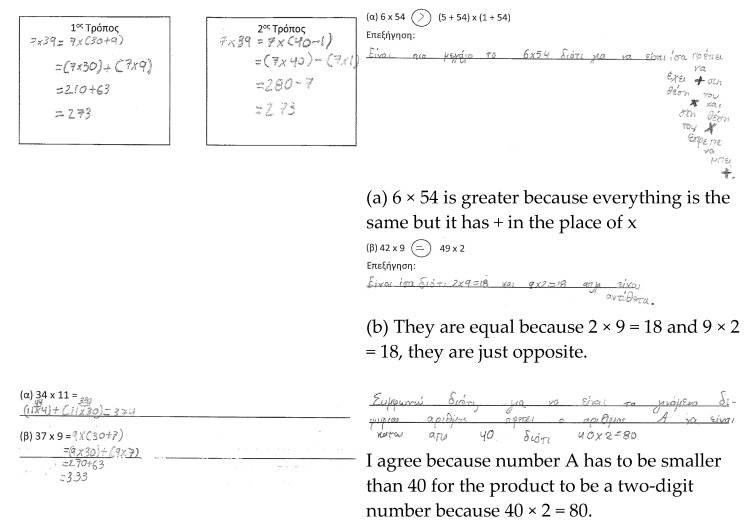
Evita’s responses.

Evita. Evita used the procedure fluently to find the product in the RT (Figure 9). In the AT1, she was looking for the “right place” of the addition and multiplication signs in order to decide whether the expressions are equivalent. She decided that since 2 × 9 = 9 × 2, the expression 42 × 9 must be equal to 49 × 2. In the AT2, she did not consider the given information to find the products, and instead used the procedure she knew (i.e., analyzing the bigger number in tens and units). Her fluency in reproducing the procedure was noticeable, but she did not show any flexibility in adapting the mathematical ideas to other formats. In the GRT, she identified that there is a set of numbers for which this statement would not be true. However, her reasoning was not presented in a coherent manner. Overall, Evita’s responses in the four tasks indicate developed fluency, limited flexibility, and developing reasoning.

**Figure 10:**
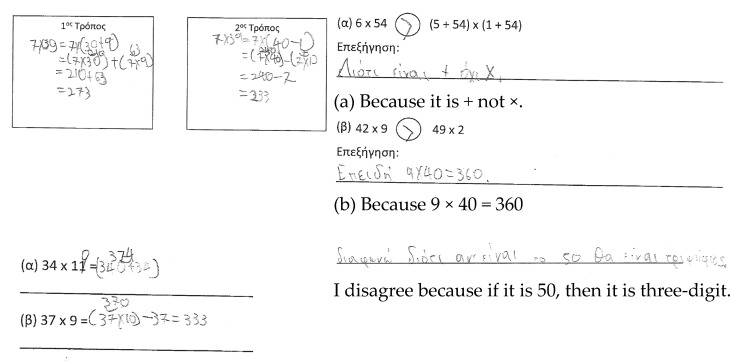
Makis’ responses.

Makis. Makis showed developing fluency in the RT due to computational mistakes in the distributive property over subtraction (Figure 10). In the AT1, he decided that 6 × 54 is greater than (5 + 54) × (1 + 54), because he identified a different sign than the expected one. In the latter expression, he seems to have made an estimation for the product. In the AT2, he used the distributive property similarly to the way in which it was used in the RT, without adapting it to the given information. Lastly, in the GRT, he provided a counterexample to refute the statement. Hence, his responses indicate developing fluency and flexibility, and developed reasoning.

## 4. Discussion

### 4.1. Vertical View of the Framework

Using the framework as a lens to design tasks and interpret students’ responses is intended to give the teacher an overall idea of the level of the class before delving into further analysis of each student’s level of competency. The framework aims to present an approach that is integrated along the continuum of teaching, learning, and assessment. Specifically, during or after the introduction of a new mathematical idea in a mathematics lesson or in a series of lessons, the teacher could use assessment tasks based on the framework to elicit evidence and interpret students’ responses. The interpretation of students’ responses could then guide the preparation and implementation of the next lesson(s). It is not necessary to offer several tasks within each of the three types of task. The number of tasks per type depends on what aspects of the taught mathematical ideas the teacher aims to assess, and in what ways these aspects are entailed in the designed tasks. In this paper, we focus on students’ written work. However, the framework is not incapable of being used during informal observations of students’ work, or during talk in the classroom.

### 4.2. Horizontal View of the Framework

Central to the framework is the role of the teacher and the previous learning opportunities in classroom. The expected processes (i.e., recall from memory, application, and generation) are framed by the contextual familiarity (i.e., students’ familiarity with the format of the task and the work procedure). A drawback of large-scale assessment is that it misses the qualitative insights on which classroom assessment could rely in order to characterize students’ emergent ideas, and the ways in which students could improve [29].

The evidence herein from students’ written responses could be viewed from different perspectives and for different purposes. For example, some could focus more on cognitive difficulties, while others focus more on the level of engagement with important mathematical processes (e.g., representing, modelling, connecting). We do not suggest that different perspectives are contradictory—at times they are complementary. To address this concern, the proposed framework presents a selected competency for each type of task, which is aligned with the expected processes and contextual familiarity. The analysis of students’ responses and the language to characterize the responses are aligned with the selected competencies. The characterizations indicate whether the student has reached a satisfying level (e.g., “developed” flexibility), whether the student is still developing the competency (e.g., “developing” flexibility), or whether the response does not provide evidence that the student is developing the competency (e.g., “limited” fluency). The framework is operationalized for the classroom teachers to inform them about students’ learning in a timely manner, in order that they might use the evidence to plan instructional adjustments.

### 4.3. Instructional Adjustments

The field of classroom assessment should focus more on how to move directly from the evidence about students’ understanding to the description of appropriate teaching actions [36]. The framework aligns the design of tasks with the analysis of students’ responses in order to set the grounds for developing hypotheses about the respective alignment with instructional adjustments.

Characterizing the level of students’ fluency could suggest possible actions for the classroom teacher. The case of “developing fluency” (Lina and Makis) indicates that further opportunities for practice could be provided in order to attend more to the series of steps, and to precision in calculations. Since students are expected to recall the mathematical idea from memory, opportunities to enhance this recall could be valued. Further study is needed in order to explore how much practice should be provided, and in what intervals. These answers might vary according to the mathematical idea(s) under study. The case of “limited fluency” is rather puzzling. If a student does not reproduce the taught mathematical idea, then student characteristics and teaching approaches should be studied further.

The cases of “developing flexibility” (Manolis and Eleonora) and “limited flexibility” (Evita and Lina) suggest that features of the tasks were not taken into consideration by the students in order to adapt the taught mathematical idea. Hence, timely feedback and focusing on the features of the tasks could enhance their learning [42]. In addition, the teaching opportunities could be infused with a variety of formats across the mathematical ideas.

For “developing reasoning” (Evita) and “limited reasoning” (Lina and Manolis), a useful approach might be the development of classroom discussions in which students are asked to persuade their classmates about their line of thought. Scholars suggest the use of prompted self-explanation and accountable talk for the learning processes of understanding and sense-making [41]. Nevertheless, further research is needed in order to provide insight into effective instructional adjustments. We agree that different processes would require different instructional adjustments. It is more likely that using the same examples with different numbers would make students better at reproducing than applying or reasoning.

### 4.4. Limitations

The framework is a starting point to discuss and elaborate further on the interplay between the design of assessment tasks and the analysis of students’ responses. Statistical analysis from various classroom settings could provide further insight. Additionally, the framework could be used and be adapted to other educational contexts and grade levels. It would be interesting to explore whether the identified processes, the contextual familiarity, the competencies, and the characterization of students’ responses are applicable and meaningful to other content areas. We anticipate that the proposed framework may have much greater validity for primary teachers, since we relied on several topics of primary mathematics for its development. The three types of processes are widely used in mathematics education and beyond. However, further research is needed in order to explore in what ways the three competencies and the characterizations of students’ responses are perceived and applied by the classroom teachers.

Moreover, it would be useful to explore the extent to which the proposed framework might be relevant in settings that use a different textbook than the one on which the framework was developed, or in settings that rely on a varied set of instructional resources rather than a textbook. We relied on a textbook series that is organized per mathematical topic. Hence, an adaptation of the framework would be needed in order to use it alongside a textbook series that is organized per mathematical process. Regarding the use of varied instructional resources, it is anticipated that the adaptation of the “expected formulation of tasks” for assessment tasks would support the application of the framework to such settings. The design of tasks and the interpretation of students’ responses rely extensively on the anticipated path based on what preceded in the classroom context, which determines the contextual familiarity (i.e., task format and work procedure), irrespective of the number of instructional resources used. Furthermore, it would be purposeful to explore the instructional adjustments in real classroom settings based on the hypotheses drawn from the framework, and how these adjustments relate to students’ learning.

## 5. Conclusions

There is evidence that classroom assessment for formative purposes has the potential to improve students’ learning [4,7]. This is a timely issue that needs to be further explored by relying on empirical evidence and systematic research. However, its effective implementation in classrooms is still in the early stages. In this paper, we move a step forward by presenting a framework that captures the interplay of the design of mathematics assessment tasks and the analysis of students’ responses along the continuum of teaching, learning, and assessment. The proposed framework provides an operational tool for the purpose of classroom assessment; it aims to provoke research that would develop insight into meaningful evidence for enhancing students’ learning of mathematics, and to set the grounds for systematically exploring instructional adjustments.

## Figures and Tables

**Table 2:** Framework for classroom assessment tasks.

Type of Task	Expected Processes	Contextual Familiarity	Competency	Characterization of Students’ Responses
Reproduction (R)	Students engage in reproducing taught mathematical ideas.	Format: Familiar Work procedure: Familiar	Fluency	*Limited* fluency *Developing* fluency *Developed* fluency
Application (A)	Students engage in applying taught mathematical ideas.	Format: Unfamiliar Work procedure: Familiar	Flexibility	*Limited* flexibility *Developing* flexibility *Developed* flexibility
Generation and Reflection (GR)	Students engage in generating and reflecting on mathematical ideas.	Format: Unfamiliar Work procedure: Unfamiliar	Reasoning	*Limited* reasoning *Developing* reasoning *Developed* reasoning

**Table 3:** Characterization of students’ responses.

Types of Tasks	Characterization of Students’ Responses	Description of the Rubric
Reproduction	Limited fluency	The student does not seem able to recall the taught mathematical idea from memory.
	Developing fluency	The student can recall the taught mathematical idea from memory, but could become more consistent.
	Developed fluency	The student can recall the taught mathematical idea directly and consistently from memory.
Application	Limited flexibility	The student does not seem able to adapt the taught mathematical idea.
	Developing flexibility	The student can coordinate the existing learning experiences to make inferences as to how to use the taught mathematical idea, but there is evidence of fragmentation.
	Developed flexibility	The student applies the taught mathematical idea in a coherent and robust manner.
Generation and Reflection	Limited reasoning	The student does not seem able to explicate the reasoning.
	Developing reasoning	The student can coordinate the set of assertions in ways that reach a conclusion and make the reasoning explicit, but there are chunks missed or interferences.
	Developed reasoning	The student presents a complete reasoning.

**Table 4:** Vertical perspective.

Type of Task	Characterization of Students’ Responses	Number of Students
Reproduction	Limited fluency	0
	Developing fluency	8
	Developed fluency	13
	Total	21
Application	Limited flexibility	9
	Developing flexibility	7
	Developed flexibility	5
	Total	21
Generation and Reflection	Limited reasoning	13
	Developing reasoning	2
	Developed reasoning	6
	Total	21
